# Supplementary material for: Isolated zero field sub-10 nm skyrmions in ultrathin Co films
Source: Nat Commun. 2019 Aug 23;10:3823. doi: 10.1038/s41467-019-11831-4 (PMC6707282; doi:10.1038/s41467-019-11831-4)
Supplement: Supplementary file 1 — Supplementary Informaion [file 41467_2019_11831_MOESM1_ESM.pdf]

Supplementary Information for

Isolated zero field sub-10 nm skyrmions in ultrathin Co films

Sebastian Meyer[1,*], Marco Perini[2,*], Stephan von Malottki[1], André Kubetzka[2], Roland Wiesendanger[2], Kirsten von Bergmann[2], and Stefan Heinze[1]

[1]Institute of Theoretical Physics and Astrophysics, Christian-Albrechts-Universität zu Kiel, Leibnizstrasse 15, 24098 Kiel, Germany

[2]Department of Physics, University of Hamburg, Jungiusstrasse 11, 20355 Hamburg, Germany

* These authors contributed equally to this work.

Supplementary Figure 1: Stacking assignment of the Rh.

Supplementary Figure 2: Lateral variations of topography and dI/dU signal.

Supplementary Figure 3: Field dependence of domain walls and skyrmions in Rh_{hcp}/Co/Ir(111).

Supplementary Figure 4: Simulated TMR and NCMR signals: line sections across a skyrmion.

Supplementary Figure 5: High-resolution zero magnetic field skyrmion.

Supplementary Figure 6: Energy dispersions of Rh/Co/Ir(111) including two models of calculating magnetic interaction parameters.

Supplementary Figure 7: Energy dispersion of flat spin spirals for Co/Ir(111), Rh/Co/Ir(111) and Co/Rh/Ir(111).

Supplementary Figure 8: Skyrmion collapse via chimera annihilation mechanism at zero field.

Supplementary Table 1: DFT values for exchange, Dzyaloshinskii-Moriya interaction and magnetocrystalline anisotropy energy for Rh_{fcc}/Co/Ir(111).

Supplementary Table 2: DFT values for exchange, Dzyaloshinskii-Moriya interaction and magnetocrystalline anisotropy energy for Rh_{hcp}/Co/Ir(111).

Supplementary Table 3: Relaxed interlayer distances for Rh/Co/Ir(111) obtained via DFT.

Supplementary Note 1: Details on the STM simulations.

Supplementary Note 2: Determination of magnetic interaction parameters via DFT.

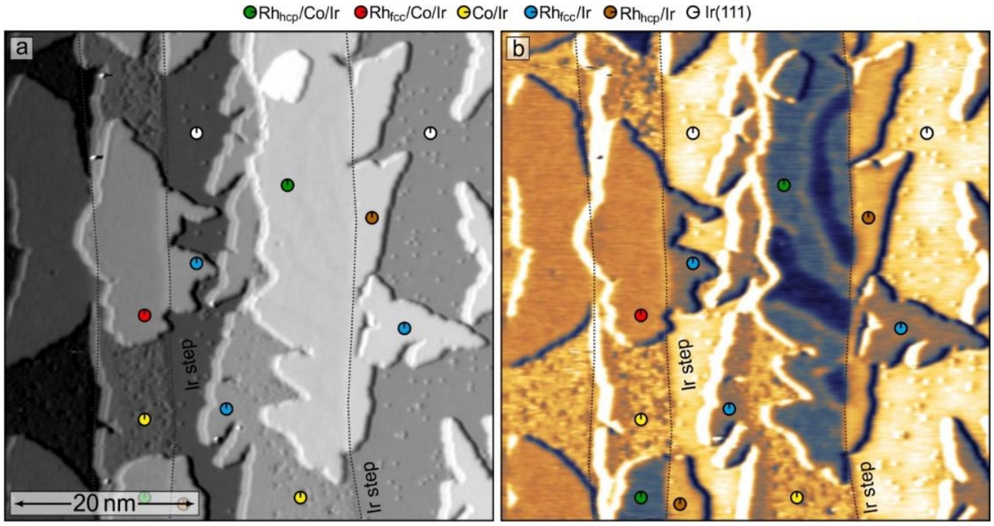

Supplementary Figure 1 | Stacking assignment of the Rh. a, Constant-current topography and **b,** simultaneously acquired map of spin-resolved differential conductance (dI/dU) of a typical sample of about 0.4 atomic layers of Rh on about 0.5 atomic layers of fcc-stacked Co on Ir(111) (measurement parameters: U = -400 mV, I = 800 pA, B = 0 T, T = 4.2 K, Cr-bulk tip; the topography is partially differentiated). Rh grows on the bare Ir(111) both as isolated islands and from the Co step edges. For the Rh/Ir areas a two-stage dI/dU signal is found, see blue and brown circles, which correspond to the two possible stackings. In one Rh-stacking the islands have irregular edges and a more dendritic shape (indicated by light blue circles in the Figure), while in the other Rh-stacking the edges are more regular and the island shape is more compact (brown circles in the Figure). Because the former stacking connects smoothly to the fcc-Co/Ir(111) we assign it to fcc-Rh/Ir(111). The other Rh/Ir(111) areas are thus in hcp stacking. Because the hcp-Rh/Ir(111) connects smoothly to one type of Rh/Co islands on the adjacent lower Ir terrace (indicated by green dots in the figure), we conclude that this type of island is the hcp-Rh/fcc-Co/Ir(111). Correspondingly, the fcc-Rh/Ir connects smoothly to the fcc-Rh/Co areas on an adjacent lower Ir terrace (red dots).

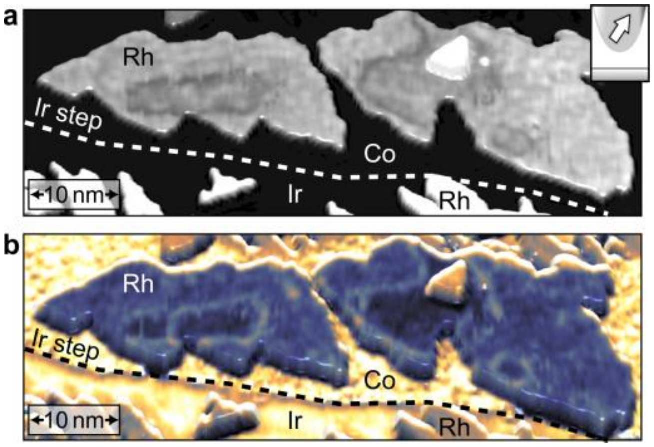

Supplementary Figure 2 | Lateral variations of topography and dI/dU signal. a, STM topography colourized with height signal and **b,** STM topography colourized with spin-resolved dI/dU map; the same area is shown in Fig. 2b (top) in the main text. The positions of the domain walls are visible in the topography due to the non-collinear magnetoresistance effect. Also, within the magnetic domains of the Rh/Co areas the topography exhibits lateral variations of the apparent height on the order of about 7 pm. We attribute this inhomogeneity to some intermixing of the two materials (measurement parameters: U = -400 mV, I = 800 pA, B = 0 T, T = 4.2 K, Cr-bulk tip).

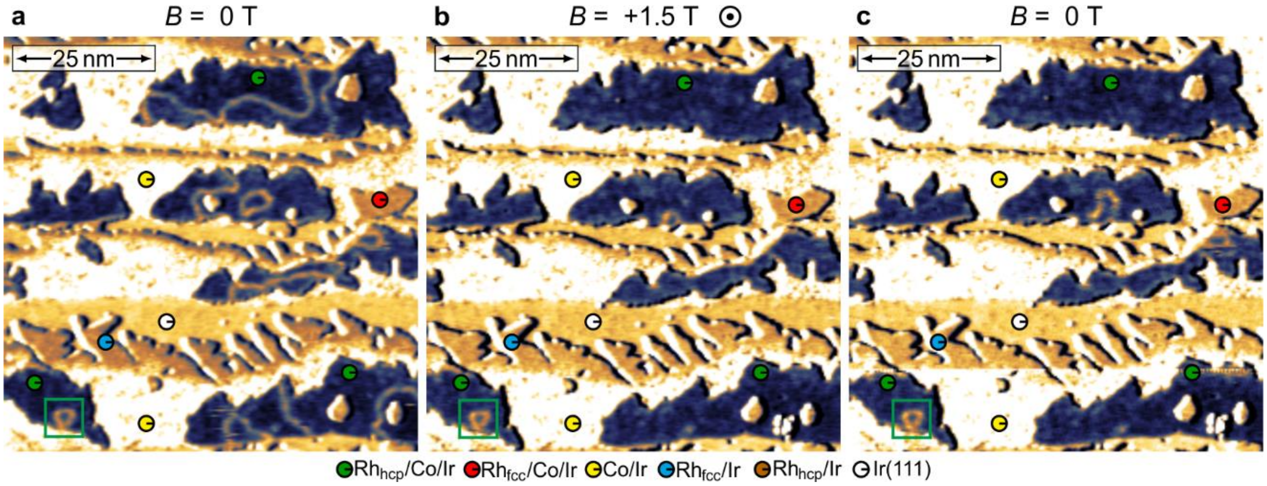

Supplementary Figure 3 | Field dependence of domain walls and skyrmions in Rh_{hcp}/Co/Ir(111). Spin-resolved differential conductance maps of a sample area with several hcp-stacked Rh islands on Co/Ir(111) (measurement parameters: U = -250 mV, I = 800 pA, T = 4.2 K, Cr-bulk tip). **a,** In zero magnetic field several domain walls are imaged via the NCMR; in addition, there is an isolated skyrmion in the bottom left corner (see box). **b,** Upon the application of an out-of-plane magnetic field of 1.5 T the majority of the domain walls are annihilated, while the skyrmion seems unaffected. **c,** After the field is brought back to zero, some domain wall movement is observed.

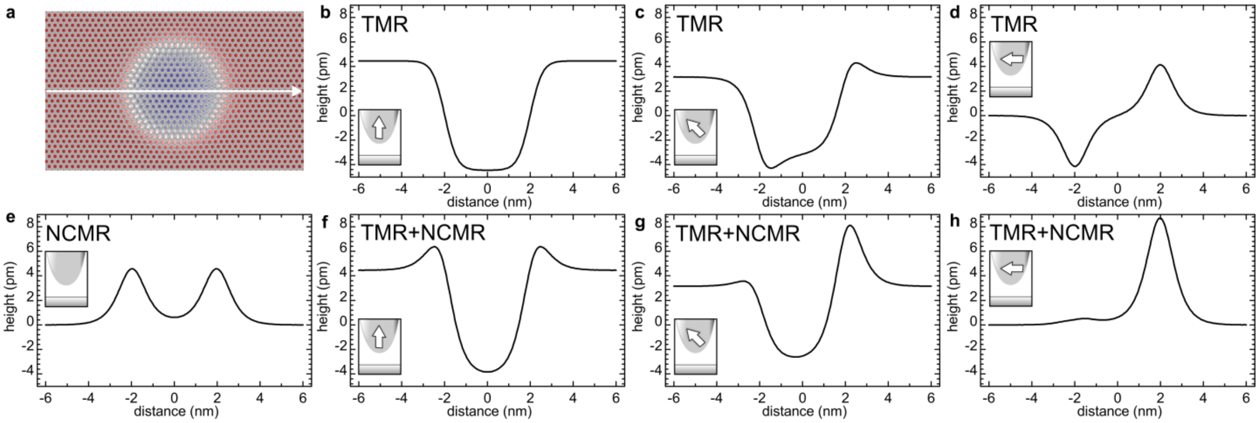

Supplementary Figure 4 | Simulated TMR and NCMR signals: line sections across a skyrmion. a, Atomic spin configuration of a (right-rotating) skyrmion, using w = 1 nm and c = 2 nm. **b,c,d,** Simulated TMR line sections across the skyrmion for a magnetic tip with a magnetization direction of 0°, 45° and 90° relative to the out-of-plane direction (see insets) using $\gamma_P = 0.1$. Here, the height signal is shown, which is nevertheless equivalent to a current or dI/dU signal as it is calculated from the simulated constant height current in linear approximation. Panel b and c qualitatively reflect the line sections in Fig. 2c, as there the NCMR signal is small. **e,** Simulated NCMR line section across the skyrmion for $\gamma_N = 0.3$, a situation similar to Fig. 3b. **f,g,h,** Simulated line sections for $\gamma_P = 0.1$ and $\gamma_N = 0.3$, with tip magnetization directions as depicted.

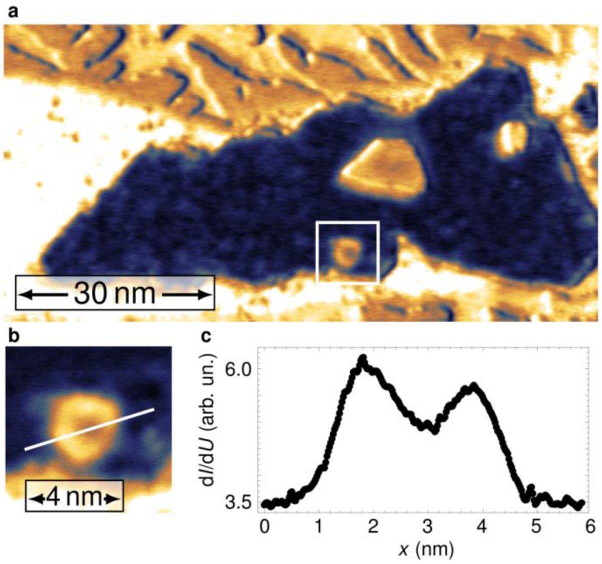

Supplementary Figure 5 | High-resolution zero field skyrmion. a, Differential conductance map showing an isolated skyrmion in zero magnetic field (measurement parameters: U = -250 mV, I = 800 pA, B = 0 T, T = 4.2 K, Cr-bulk tip). The object is visible due to the NCMR contrast. **b,** Closer view of the skyrmion. **c,** dI/dU signal across the skyrmion, see white line in **b**. The diameter of this skyrmion is about 2.2 nm.

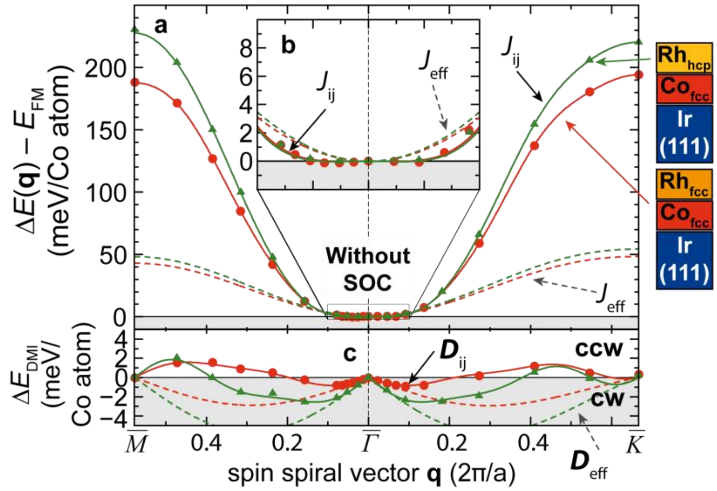

Supplementary Figure 6 | Energy dispersions of Rh/Co/Ir(111) including two models of calculating magnetic interaction parameters. a, Energy dispersion without spin-orbit coupling (SOC). **b,** Zoom of a region around $\mathbf{q} \to \mathbf{0}$. **c,** Energy contribution due to SOC. The points represent the DFT calculations, the solid lines show fitted curves using magnetic interaction parameters beyond nearest neighbours $\boldsymbol{J_{ij}}, \mathbf{D_{ij}}$ from Supplementary Tables 1 and 2 while the dashed lines correspond to effective nearest neighbour parameters $\boldsymbol{J}_{\mathbf{eff}}, \mathbf{D}_{\mathbf{eff}}$.

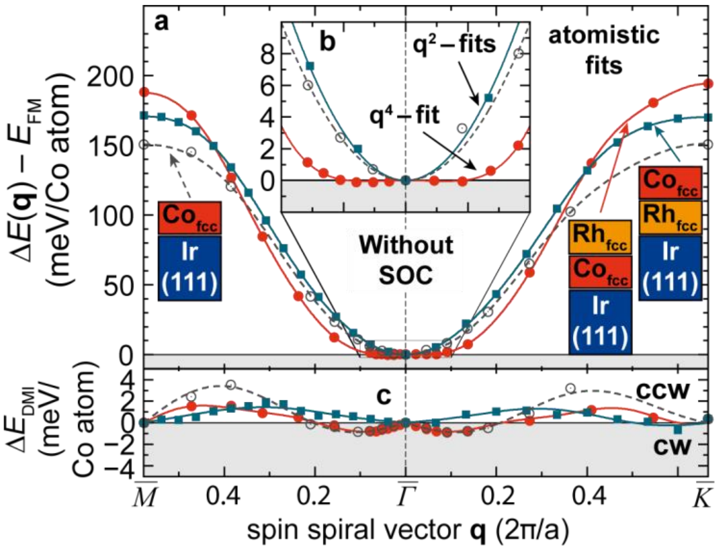

Supplementary Figure 7 | Energy dispersion of flat spin spirals for Co/Ir(111), Rh/Co/Ir(111) and the inverted layer sequence Co/Rh/Ir(111). a, Energy difference between spin spiral states $E(q)$ with respect to the ferromagnetic (FM) state without spin-orbit coupling (SOC). The $\bar{\Gamma}$-point corresponds to the FM state, the $\bar{M}$-point to the row-wise antiferromagnetic (AFM) state and the $\bar{K}$-point to the 120° Néel state. The points represent the DFT calculations while the lines are the fits to the Heisenberg exchange interactions beyond nearest neighbours. **b,** Zoom to a region around the FM area ($\bar{\Gamma}$-point) of **a**, where the lines represent a simple q^2 fit in the case of Co/Ir(111) and Co/Rh/Ir(111) and a fit including a q^4 term in the case of Rh/Co/Ir(111). **c,** Energy contribution due to the Dzyaloshinskii-Moriya interaction (DMI) for the three systems. The points show the calculated values using first order perturbation theory and the lines are the fits to the DMI beyond nearest neighbours. Positive (negative) values correspond to a counterclockwise (clockwise) rotation preferred by the DMI.

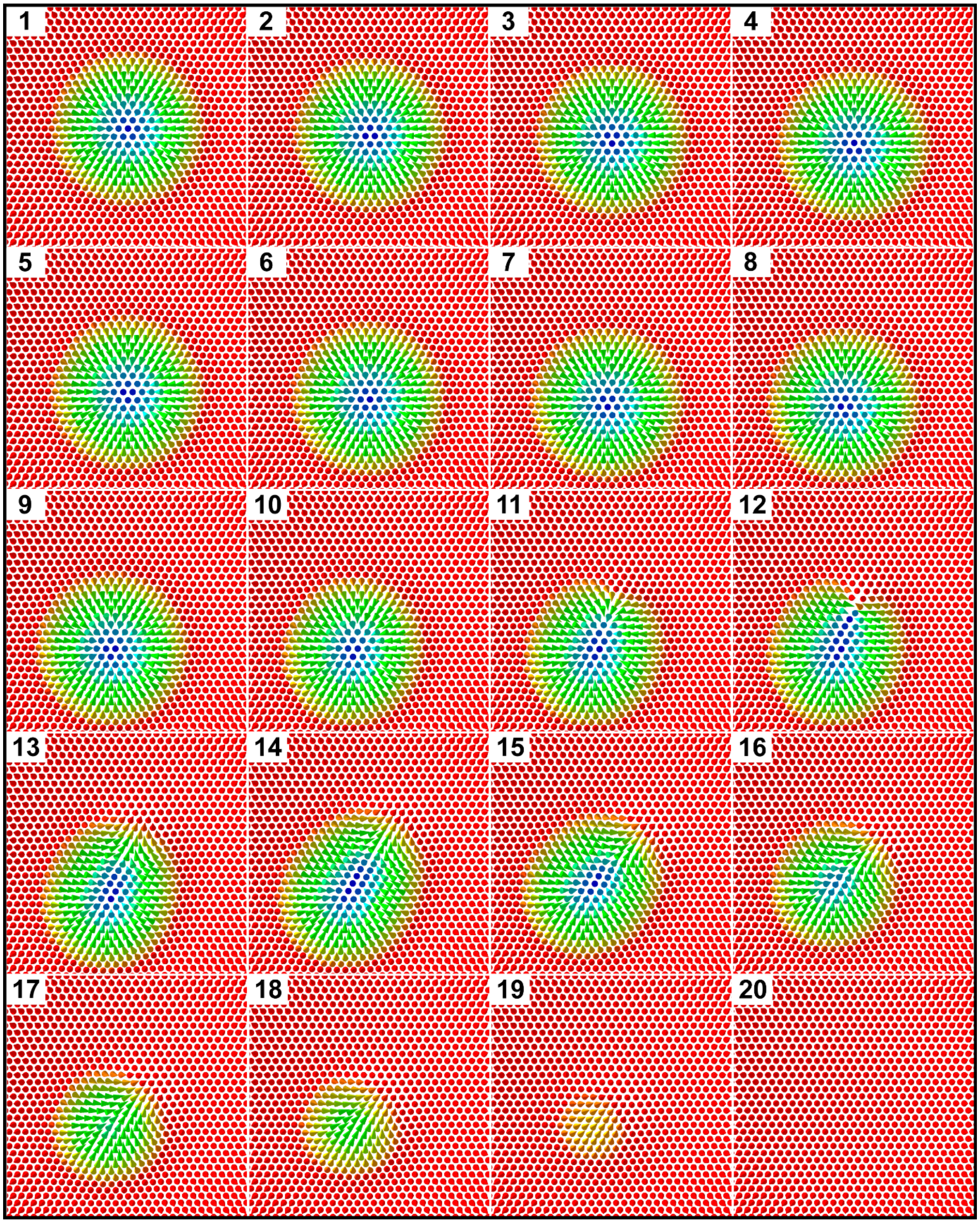

Supplementary Figure 8 | Skyrmion collapse via chimera annihilation mechanism at zero field. The initial isolated skyrmion state (image 1) first drifts within the simulation box and is then slightly deformed (image 11) before the saddle point (image 12) is reached. The chimera skyrmion with zero topological charge is formed (image 13), which easily collapses into the ferromagnetic final state (image 20). Colours denote the out-of-plane magnetization component. Red cones point upwards and blue cones point downwards. Note that only a small area around the skyrmion stabilized in an 70x70 spin lattice is shown.

J_1	J_2	J_3	J_4	J_5
$+25.1802$	$+0.2538$	-2.7064	-0.6335	-0.2375
J_6	J_7	J_8	J_9	J_{10}
$+0.0950$	$+0.0162$	$+0.2727$	$+0.0299$	-0.2814
D_1	D_2	D_3	D_4	D_5
-0.2935	$+0.1064$	$+0.2944$	-0.0330	-0.0217
D_6	D_7	K	D_{eff}	J_{eff}
-0.0383	$+0.0876$	-1.1660	$+0.8333$	$+5.3760$

Supplementary Table 1 | DFT values for exchange, Dzyaloshinskii-Moriya interaction and magnetocrystalline anisotropy energy for Rh_{fcc}/Co/Ir(111). Exchange constants J_i and DMI constants D_i for i-th nearest neighbours as well as magnetocrystalline anisotropy energy K are given in meV/Co atom. Positive values of D_i represent a clockwise rotation preferred by the DMI, negative values of K indicate an out-of-plane anisotropy. Four digits are given for all parameters as used in our atomistic spin dynamics simulations in order to allow reproducibility of our results by other spin dynamics codes e.g. Spirit Code [7] or UppASD [8].

J_1	J_2	J_3	J_4	J_5
$+29.2169$	$+0.8550$	-4.0687	-0.4136	-0.5804
J_6	J_7	J_8	J_9	J_{10}
-0.1268	$+0.0055$	$+0.0468$	$+0.0478$	-0.1586
D_1	D_2	D_3	D_4	D_5
$+0.1303$	$+0.3816$	$+0.3741$	-0.0277	-0.2388
D_6	D_7	K	D_{eff}	J_{eff}
-0.0564	$+0.1784$	-1.6350	$+1.6345$	$+6.0300$

Supplementary Table 2 | DFT values for exchange, Dzyaloshinskii-Moriya interaction and magnetocrystalline anisotropy energy for Rh_{hcp}/Co/Ir(111). Exchange constants J_i and DMI constants D_i for i-th nearest neighbours as well as magnetocrystalline anisotropy energy K are given in meV/Co atom. Positive values of D_i represent a clockwise rotation preferred by the DMI, negative values of K indicate an out-of-plane anisotropy. For the spin dynamics simulations of Fig. 4 in the main text, we applied $J_i, D_i/2$ and K. Four digits are given for all parameters as used in our atomistic spin dynamics simulations in order to allow reproducibility of our results by other spin dynamics codes e.g. Spirit Code [7] or UppASD [8].

	$d_{\text{Rh-Co}}$	$d_{\text{Co-Ir(S)}}$	$d_{\text{Ir(S)-Ir(S-1)}}$	d_{bulk}
Rh_{fcc}/Co/Ir(111)	2.057	2.150	2.304	2.204
Rh_{hcp}/Co/Ir(111)	2.052	2.126	2.334	2.204

Supplementary Table 3 | Relaxed interlayer distances for Rh/Co/Ir(111) obtained via DFT. **The interlayer distances d (Å) are shown for the relaxed layers and the Ir bulk. We applied the generalized gradient approximation (GGA, pbe) [6] of the exchange correlation (xc-) potential.**

Supplementary Note 1: Details on the STM simulations.

The magnetic skyrmions observed in the dI/dU maps of Fig. 1 are well reproduced by simulated STM images. Input for such a simulation is the magnetic state of the sample, which is modelled by a 2D arrangement of spins $\mathbf{S}_i$ located at the atomic positions $\mathbf{r}_\mathbf{i}$. The signal, i.e. the current or the dI/dU intensity, is assumed to decay exponentially with distance from each atom. Then, for a given tip-sample distance z_0, the signal for each point of the STM image is calculated as the sum of the contributions from all spins [3] within a certain distance from the tip position $\mathbf{r}_T$, with a typical cut-off distance of $3z_0$. The resulting dI/dU signal at the tip position is then:

$$\frac{dI}{dU}(\mathbf{r}_T) = \sum_{i \in M} \left(\frac{dI}{dU}\right)_i^{loc} e^{-2k|\mathbf{r}_T - \mathbf{r}_i|}$$

where $k = \sqrt{\frac{2m_e\phi}{\hbar^2}}$ is the decay constant and ϕ is the sample work function. M indicates the set of spin indices within the cut-off distance. $(dI/dU)_i^{loc}$contains the bias voltage dependent contributions to the differential conductance from the non-spin-polarized collinear magnetic state $(dI/dU)_0$, the tunnel magnetoresistance (TMR), and the non-collinear magnetoresistance (NCMR):

$$\left(\frac{dI}{dU}\right)_i^{loc} = \left(\frac{dI}{dU}\right)_0 (1 + \gamma_P\, \mathbf{S}_i \cdot \mathbf{m}_T + \gamma_N \bar{\alpha}\,)$$

where $\mathbf{m}_T$ is the tip magnetization direction, and γ_P and γ_N are the pre-factors determining the strength of TMR and NCMR, respectively. Here γ_P is the spin-polarization at the particular bias voltage and the signal intensity scales with the projection of tip and local sample magnetization due to the TMR. γ_N is an empirical prefactor to capture the NCMR contribution, which is modelled to be proportional to $\bar{\alpha}$, the average angle between the direction of the spin $\mathbf{S}_i$ and those of its six nearest neighbours [4,5]. Examples of simulated profiles across a skyrmion for each of these two contributions and their combination is shown in Supplementary Figure 4.

The domain walls were modelled according to a standard 180° domain wall, $\cos\varphi = \tanh(\frac{x}{w/2})$, of width w, the magnetic skyrmions were modelled by a circular standard 180° domain wall of width w and radius

c [1]. The skyrmion diameter is defined as the distance between opposite in-plane magnetizations. The work function was set to $\phi = 4.8$ eV and the tip sample distance was $z_0 = 0.8$ nm.

For the simulations of the two oppositely magnetized skyrmions in Fig. 2 the following parameters were used:
$\mathbf{m}_T = (\cos\varphi \sin\theta, \sin\varphi \sin\theta, \cos\theta)$, with φ = 110° and θ = 48°.
For Fig. 2c: $\gamma_P = 0.3, \gamma_N = 0.064$. For Fig. 2d: $\gamma_P = 0.075, \gamma_N = 0.302$.
For the skyrmion in the upper images (indicated in magenta): $w = 0.9$ nm, $c = 2.15$ nm ($d = 4.3$ nm). For the skyrmion in the lower images (indicated in green): $w = 1.0$ nm, $c = 1.77$ nm ($d = 3.5$ nm).

For the simulations of Fig. 3 the TMR contribution was neglected and $\gamma_N = 0.452$.
The magnetic skyrmion of Fig. 3b is modelled by: $w = 0.8$ nm, $c = 1.42$ nm ($d = 2.8$ nm).

Supplementary Note 2: Determination of magnetic interaction parameters via DFT.

We obtain the exchange constants J_{ij} of the Heisenberg model by calculating the energy dispersion of homogeneous, flat spin spirals via DFT as implemented in the FLEUR code [9,10]. Spin spirals can be described by the spin spiral vector $\mathbf{q}$ determining the propagation direction of the spiral and the angle between two adjacent spins along the spiral. The $\mathbf{q}$-vector is a vector in reciprocal space and is chosen along the high symmetry directions of the hexagonal Brillouin zone (BZ): $\bar{M} - \bar{\Gamma} - \bar{K}$. The magnetic moment of atom i at position $\mathbf{R}_i$ is given by $\mathbf{M}_i = M[\cos(\mathbf{q} \cdot \mathbf{R}_i) \sin\theta, \sin(\mathbf{q} \cdot \mathbf{R}_i) \sin\theta, \cos\theta]$. Here, θ is the opening angle for conical spin spirals and in the case of flat spin spirals it is 90°.

Neglecting spin-orbit coupling (SOC), left and right rotating spin spirals are energetically degenerate. Since there is no preferred magnetization direction without SOC, the generalized Bloch theorem can be applied to self-consistently calculate the energy differences between different spin spiral states ($\mathbf{q} \neq 0$) and the ferromagnetic state (FM state, $\mathbf{q} = 0$) within the chemical unit cell. The resulting energy dispersion $E(\mathbf{q})$ is mapped to the Heisenberg model

$$\mathcal{H} = -\sum_{ij} J_{ij}(\mathbf{m}_i \cdot \mathbf{m}_j) \qquad (1)$$

where $\mathbf{m}_i = \frac{\mathbf{M}_i}{M_i}$ is the unit vector of the magnetic moment of atom i.

In Fig. 4a of the main text, the energy dispersion close to the $\bar{\Gamma}$-point is very flat. At the same time the energy difference between the FM ($\bar{\Gamma}$), the row-wise antiferromagnetic ($\bar{M}$) and the Néel ($\bar{K}$) state (with angles of 120° between neighbouring moments) is large, which requires a fit including ten neighbours to describe the behaviour of the full energy dispersion. The resulting values for $J_1 \dots J_{10}$ are presented in Supplementary Tables 1 and 2 for Rh_{fcc}/Co/Ir(111) and Rh_{hcp}/Co/Ir(111), respectively.

When an energy dispersion behaves like q^2 for $q \to 0$ as in the standard micromagnetic model, it is possible to fit this range with an effective nearest neighbour interaction J_{eff}. However, in Rh/Co/Ir(111) the resulting J_{eff} varies significantly with the fitting region around the FM state between 1 and 10 meV/Co atom. In order to get a reasonable value for J_{eff}, we evaluate domain wall widths w from spin-dynamics simulations where we included all atomistic magnetic parameters beyond nearest neighbours of Supplementary Tables 1 and 2. We determine the effective nearest neighbour exchange interaction $J_{\text{eff}} = Kw^2/6a^2$ [11], where K is the uniaxial magnetocrystalline anisotropy and a the lattice constant of

the Ir(111) plane, which completes the magnetic parameters in Supplementary Tables 1 and 2. The resulting energy dispersion is presented in Supplementary Figure 6, and it becomes apparent that they differ strongly from the calculated DFT values. Consequently, the standard micromagnetic model with an exchange stiffness based on a q^2 energy dependence is insufficient to describe the dispersion around the ground state (cf. Fig. 4b in the main text). Although Co is a strong FM and normally described very well by the conventional micromagnetic model, here the next order of the Taylor expansion (q^4) contributes significantly. We recover the q^2 energy dependence close to the $\bar{\Gamma}$-point upon interchanging the Co and the Rh layers, i.e. for Co/Rh/Ir(111) (see Supplementary Figure 7). This also indicates that intermixing of Co and Rh atoms at the interface (which is 100% for Co/Rh/Ir(111)) will lower the exchange frustration thereby supporting the FM state.

The degeneracy of left and right rotating spin spiral states is lifted upon including SOC. Two additional energy contributions will occur due to the presence of SOC: the magnetocrystalline anisotropy energy (MAE) and the antisymmetric exchange interaction, known as the Dzyaloshinskii-Moriya interaction (DMI). The DMI requires a broken inversion symmetry, which is given by the interface and the surface of all films. If SOC is included, the generalized Bloch theorem cannot be used to calculate spin spirals in the chemical unit cell. However, since SOC is typically small compared to the total energy in the system, we treat SOC in first-order perturbation theory starting from self-consistent spin spiral calculations [12]. This approximation has been checked previously and the deviations due to using first-order perturbation theory are up to 20% depending on the spin spiral period [13]. The resulting energy contribution due to SOC is mapped onto the DMI,

$$\mathcal{H}_{\mathrm{DMI}} = -\sum_{ij} \mathbf{D}_{ij}(\mathbf{m}_i \times \mathbf{m}_j) \qquad (2)$$

where $\mathbf{D}_{ij}$ is the DMI vector which determines the strength and the sign of the DMI. There are two possibilities to fit the model to the DFT values. We can determine the gradient of the SOC contribution around the ground state, since the sine behaviour of the DMI is linear around $\mathbf{q} \to 0$. The result of this procedure is given by $\mathbf{D}_{\mathrm{eff}}$ in Supplementary Tables 1 and 2. However, the behaviour of the SOC contributions of Fig. 4c in the main text and Supplementary Figure 6 is not represented by a simple sine. Instead, we have to include 7 neighbours to achieve a good description of the DMI leading to a frustration of the DMI similar to the exchange interaction.

The second energy contribution due to SOC is the magnetocrystalline anisotropy energy (MAE). After self-consistent scalar-relativistic calculations, we apply SOC in the out-of-plane (⊥) and in-plane (‖) direction and use the force theorem [14] to obtain the MAE which is defined as the total energy difference between the two magnetization directions: $K = E_{\perp} - E_{\parallel}$. We restrict ourselves to the uniaxial anisotropy,

$$E_{\mathrm{MAE}} = \sum_{i} K(\mathrm{m}_i^z)^2 \qquad (3)$$

which is a good approximation for our ultrathin film systems.

Supplementary References

[1] Romming, N., Kubetzka, A., Hanneken, C., von Bergmann, K. & Wiesendanger, R. Field-dependent size and shape of single magnetic skyrmions. *Phys. Rev. Lett.* **114**, 177203 (2015).

[2] Bogdanov, A. & Hubert, A. The properties of isolated magnetic vortices. *Phys. Stat. Sol. (b)* **186**, 527-543 (1994).

[3] Heinze, S. Simulation of spin-polarized scanning tunneling microscopy images of nanoscale non-collinear magnetic structures. *App. Phys. A* **85**, 407-414 (2006).

[4] Hanneken, C., Otte, F., Kubetzka, A., Dupé, B., Romming, N., von Bergmann, K., Wiesendanger, R. & Heinze, S. Electrical detection of magnetic skyrmions by tunneling non-collinear magnetoresistance. *Nature Nanotech*. **10**, 1039-1042 (2015).

[5] Kubetzka, A., Hanneken, C., Wiesendanger, R. & von Bergmann, K. Impact of the skyrmion spin texture on magnetoresistance. *Phys. Rev. B* **95,** 104433 (2017).

[6] Zhang, Y. & Yang, W. Comment on "Generalized Gradient Approximation Made Simple". *Phys. Rev. Lett.* **80**, 890-890 (1998).

[7] https://iffwiki.fz-juelich.de/index.php/Spirit

[8] https://github.com/UppASD/UppASD

[9] www.flapw.de

[10] Kurz, Ph., Förster, F.,Nordström, L., Bihlmayer, G., & Blügel, S. Ab initio treatment of noncollinear magnets with the full-potential linearized augmented plane wave method. *Phys. Rev. B* **69**, 024415 (2004).

[11] Perini, M., Meyer, S., Dupé, B., von Malottki, S., Kubetzka, A., von Bergmann, K., Wiesendanger, R. & Heinze, S. Domain walls and Dzyaloshinskii-Moriya interaction in epitaxial Co/Ir(111) and Pt/Co/Ir(111). *Phys. Rev. B* **97**, 184425 (2018)

[12] Heide, M., Bihlmayer, G., & Blügel, S. Describing Dzyaloshinskii-Moriya spirals from first-principles. *Physica B* **404**, 2678 (2009).

[13] Meyer, S., Dupé, B., Ferriani, P. & Heinze, S. Dzyaloshinskii-Moriya interaction at an antiferromagnetic interface: First-principles study of Fe/Ir bilayers on Rh(001). *Phys. Rev. B* **96**, 094408 (2017).

[14] Oswald, A., Zeller, R., Braspenning, P. J. & Dederichs, P. H. Interaction of magnetic impurities in Cu and Ag. *J. Phys. F* **15**, 193 (1985)
